# Aggression in Low Functioning Children and Adolescents with Autistic Disorder

**DOI:** 10.1371/journal.pone.0014358

**Published:** 2010-12-21

**Authors:** Guillaume Bronsard, Michel Botbol, Sylvie Tordjman

**Affiliations:** 1 Maison Départementale de l'Adolescent et Centre Médico-Psycho-Pédagogique (Conseil Général des Bouches-du-Rhône), Laboratoire de Santé Publique (EA3279) de la Faculté de Médecine de la Timone, Marseille, France; 2 Ecole des Psychologues Praticiens, Paris Catholic University, Société Psychanalytique de Paris, Paris, France; 3 Laboratoire Psychologie de la Perception, UMR 8158 CNRS, Université Paris Descartes, and Service Hospitalo-Universitaire de Psychiatrie de l'Enfant et de l'Adolescent de Rennes, Université de Rennes 1, France; Royal Children's Hospital, Queensland, Australia

## Abstract

**Background:**

Parents, caregivers and mental health professionals have often reported violence and aggression in children or adolescents with autistic disorder. However, most of these observations derived from anecdotal reports, and studies on frequency and characterization of aggression in autism remain limited. Our objective was to better characterize and understand the different types of aggressive behaviors displayed by a large group of individuals with autism in different observational situations.

**Methodology/Findings:**

The study was conducted on 74 children and adolescents with autism and 115 typically developing control individuals matched for sex, age and pubertal stage. Other-Injurious Behaviors (OIB) were assessed in three observational situations (parents at home, two caregivers at day-care, a nurse and a child psychiatrist during blood drawing) using validated scales. The frequency of OIB was significantly higher in individuals with autism compared to typically developing control individuals during the blood drawing (23% vs. 0%, *P*<0 .01). The parents observed significantly less OIB in their children than caregivers (34% vs. 58%, *P*<0.05). In addition, the most frequent concurrent behaviors occurring just before the appearance of OIB in individuals with autism were anxiety-related behaviors and excitation according to the parental as well as the caregiver observation.

**Conclusions/Significance:**

The results suggest that in a stressful situation, such as the blood drawing, individuals with autism release their stress through behaviors such as OIB, whereas typically developing individuals regulate and express their stress through cognitive skills such as mental coping strategies, symbolization skills with representation and anticipation of the stressful situation, social interaction and verbal or non-verbal communication. The findings underline also the key role of the environment in assessing OIB and developing therapeutic perspectives, with an individual who modulates his/her behavior according to the environment, and an environment that perceives this behavior and reacts to it with different tolerance thresholds according to the observers.

## Introduction

Aggression in children with autistic disorder places these children and the aggressed individuals at risk for physical injury [1], [2] while limiting their integration in community and educational activities [3]-[7]. Some authors consider these aggressive behaviors, when they are uncontrolled in young children with autistic disorder, to be a sign of poor prognosis [8], [9]. It is necessary to question first what is meant by aggression. Aggression has been defined by Buss [10] as a response that delivers noxious stimuli to another organism. The noxious stimuli may be physical or verbal. Vitiello and Stoff [11] defined later aggression as behavior deliberately aimed at inflicting physical damage to persons or property. Instruments used currently in psychiatry in order to measure aggression are based on diverse definitions of aggression. In this article we will focus on other-injurious behaviors (OIB) directed against people or objects.

The field of developmental psychology sheds some light onto the modifications of aggression in the first years of life. The peak of aggression is observed between 18 and 24 months of age and aggression decreases with time after the age of two years old [12]. Some authors [13], [14] suggest that aggression can be used in play with the child and others, allowing the development of control of this behavior. In autism, the absence of language or severe communication impairment, as well as the deficit of theory of mind (i.e., the lack of empathy and inability to represent other's mental state due to a deficit in abstraction [15]) preventing access to pretend play, may explain the lack of control of aggression and thus its persistence [16]-[18], although this interpretation is controversial [19]. This reinforces the hypothesis that persistent OIB would result more from a lack of “learning not to aggress” rather than from an excess of “learning to aggress” [13], [14], [20].

Aggressive behaviors were found to be two to three times more prevalent among children with developmental disabilities compared to typically developing children [21]. Furthermore, McClintock [22] reported that autism was a « risk marker » for aggression and disruptions to the environment when compared to non-autistic individuals with developmental disabilities. There are numerous anecdotal reports of violence and aggression in high functioning individuals with autism spectrum disorder [23]-[25], but studies of its frequency and characterization remain limited in large samples of low functioning children with autistic disorder. We have conducted the present study to better understand and characterize the different types of OIB occurring in three observational situations (parental, caregivers, blood drawing) in a large sample of low functioning children and adolescent with autistic disorder, and to compare OIB across the autistic and typically developing control groups during the blood drawing situation. This study was a part of a larger project (INSERM CRE 931009) that had as its overall objective the examination of associations between behavioral profiles and biological variables in children with autism. The larger project required a blood drawing and included, in addition to the study of aggression, an examination of the relationship between plasma endorphin and pain-related behaviors [26].

## Methods

### Participants

Children and adolescents with autism (N = 74) were recruited from French day-care facilities, and included 49 males and 25 females (mean age = 11.6 years, SD = 4.5; 32 pre-pubertal, 16 pubertal, 26 post-pubertal). Based on direct clinical observation by two independent child psychiatrists, a diagnosis of autistic disorder was made according to DSM-IV [27], ICD-10, CFTMEA [28] criteria and was confirmed by the ADI-R (Autism Diagnostic Interview-Revised) [29] ratings. Following a procedure previously described [30], we took the median value of all items belonging to the same domain of autistic impairment according to the ADI-R algorithm that is based on the 4-5 years-old period of life. This gave a score of central tendency for each of the three main domains: Total Reciprocal Social Interaction (15 items), Total Verbal/non-verbal Communication (13 items; for non-verbal patients the median score was based on 9 items), Total Stereotypies (8 items). Based on this procedure, our autism group was characterized at 4-5 years old by severe impairment in the verbal/non-verbal communication domain or the reciprocal social interaction domain, and mild impairment in the stereotypies domain. It is noteworthy that 49 out of 74 individuals with autism showed currently an absence of verbal language according to the ADI-R definition (absence of verbal language is defined as the absence of daily, functional and comprehensible use of spontaneous phrases of at least three words, including at least sometimes a verb).

Typically developing control individuals (N = 115) were recruited over a three-month period from the Reims preventive medical center, and included 75 males and 40 females (mean age = 12.7 years, SD = 5.9; 45 pre-pubertal, 27 pubertal, 43 post-pubertal). They were determined by two independent pediatricians to be free of significant psychopathology and any developmental or neurological disorders. Additionally, there was no family history of autism in the first degree relatives of individuals in the control group.

The typically developing control individuals and individuals with autism were matched on age, sex and Tanner stage of puberty. The two groups did not differ significantly with respect to age, sex, and pubertal status. All individuals in both subject groups were sleeping in their parents' house and were attending school or college for the typically developing control individuals and day-care facilities for the individuals with autism on a daily basis from about 9am to 4pm. All subjects were Caucasian, physically healthy and had no history of encephalopathy or neuroendocrinological disease. All typically developing control individuals were unmedicated, while forty-eight patients with autism were unmedicated. Fourteen patients with autism had a history of idiopathic epilepsy and were being treated with anticonvulsants. Fifteen patients with autism were receiving neuroleptics. The protocol was approved by the ethics committee of Bicêtre Hospital and written informed consent was obtained from parents.

### Behavioral and Cognitive Assessments

Cognitive functioning of autistic individuals was assessed by two psychologists using the age-appropriate Wechsler intelligence scales (WPPSI-R, WISC-R, WAIS-R) and the Kaufman K-ABC [31]. All individuals with autism were cognitively impaired (mean full scale IQ ± SD: 42.2±3.2, with range of 40-58; mean verbal IQ ± SD: 45.5±2.2; with a range of 45-57; mean performance IQ ± SD: 45.6±4.1, with a range of 45-57).

Assessments of other-injurious behavior in individuals with autism were performed using the Other-Injurious Behavior Scale (OIB scale) [32], [33]. The OIB scale was used to assess other injurious behavior (OIB) by providing quantitative ratings (scored from 1 to 7) based on frequency, severity and duration for 15 types of OIB (see **Table 1**). This scale assesses the current and lifetime OIB. It provides also a context subscale indicating the circumstances in which the OIB occurs and the concurrent behaviors occurring just before the OIB, in order to better understand the appearance of OIB.The OIB scale has been previously found to have good discriminative capacity and to be reliable (inter-rater reliability) and valid (internal and external validity) for assessment of other-injurious behaviors in autistic disorder [33].

**Table 1:** Caregiver and Parental Evaluations of different Types of Other-Injurious Behavior (OIB) and Spearman Correlations between Caregiver and Parental Evaluations of OIB in individuals with Autism (n = 74).

Type of Other-Injurious Behavior	Caregiver Evaluation	Parental Evaluation	r Spearman	*P*
1. Slapping	27 (36%)	13 (17.5%)	0.26	.02
2. Pinching/Holding others tight (with the arms)	25 (33%)	17 (23%)	0.51	<.0001
3. Explosion-scattering of objects (e.g. throwing the objects around, but not directed against others)	24 (32%)	14 (18.9%)	0.06	.59
4. Pitching into others with the head (violent and unexpected head banging, head against other's chest)	16 (21.6%)	6 (8%)	-0.01	.92
5. Scratching	11 (14.9%)	7 (9.4%)	0.15	.19
6. Tearing (e.g. clothes)	11 (14.9%)	6 (8%)	0.07	.56
7. Pulling out (hair, scab, skin)	10 (13.5%)	4 (5.4%)	0.29	.01
8. Biting	9 (12.1%)	8 (10.8%)	0.37	.0009
9. Mashing (objects or people with the feet)	7 (9.4%)	0 (0%)	-0.08	.46
10. Face attack	7 (9.4%)	2 (2.7%)	0.03	.81
11. Poking others (objects, people)	6 (8%)	2 (2.7%)	0.13	.24
12. Attacking others with objects	5 (6.7%)	5 (6.7%)	0.21	.07
13. Spitting at others	5 (6.7%)	4 (5.4%)	0.32	.005
14. Punching others	3 (4%)	3 (4%)	0.007	.90
15. Inserting objects into others' face orifices	2 (2.7%)	2 (2.7%)	0.14	.21

*Note:* Data are frequency of patients (% of group) for each type of others-injurious behavior.

A same patient can present different types of other-injurious behavior. Spearman correlations were calculated on the quantitative scores of OIB provided by the OIB scale.

Assessments of OIB were performed for individuals with autism in three different observational situations: 1) in day-care, where two caregivers independently rated OIB on a daily basis during the month preceding the blood drawing for all the individuals with autism participating to the study; 2) at home, where parents rated behavior during the same month as the caregivers; 3) during the blood drawing at a medical center, when a direct clinical observation was conducted by a nurse and child psychiatrist not belonging to the caregiver team. Typically developing control individuals were similarly assessed for the presence or absence of any types of OIB. Pubertal status was assessed in autism and control groups during the blood drawing situation by pediatricians using the Tanner scale [34]. The blood drawing was performed for 64 patients with autism at the nearest general hospital rather than at the caregiver day hospital so that the procedure was not associated with the therapeutic setting. Blood drawing for controls occurred at the Reims preventive medical center. The blood drawing followed a standardized procedure to minimize and control the possible stressful conditions. For all patients (n = 64) and controls (n = 115), parents were present during all the blood drawing and no white coats were worn in the presence of the subjects; the subjects stayed in a play room for 15 minutes before the blood drawing and all the blood drawing were performed by the same nurse, who was particularly experienced with handicapped children.

### Statistical Analysis

Group and subgroup comparisons of OIB were performed using analysis of variance (ANOVA) and two-tailed t-test. Correlations were determined by Spearman or Pearson correlation analyses. Group comparisons between individuals with autism and typically developing control individuals for OIB in the blood drawing situation were assessed by χ2 test.

## Results

### Relationships between descriptive variables and OIB

There were no significant effects of IQ, sex, age, pubertal status and medical status (neuroleptics, anticonvulsants, or all medications combined) on OIB regardless of the observational situation (parental, caregiver, blood drawing) in individuals with autism.

### Caregiver and parental evaluations of OIB in individuals with autism

Distributions of the different types of OIB according to the parental and caregiver observational situations are presented in **Table 1**. A high percentage of individuals with autism displayed OIB (caregiver evaluation: 43/74 (58%); parental evaluation: 25/74 (34%)), especially slapping, pinching-holding others tight and explosion-scattering of objects. A statistical comparison of the one-month observational situations in patients (parental, caregiver) showed that parents observed significantly (*P*<.05) less OIB (for any types of OIB) in their children than caregivers. Furthermore, a significant correlation between these two observational situations was found only for certain quantitative scores (Spearman correlations) of OIB (slapping, pinching-holding others tight, pulling out, biting, spitting at others) (see **Table 1**).

The emotional circumstances in which OIB occurred and their concurrent behaviors are presented in **Table 2** for the caregiver assessment and in **Table 3** for the parental assessment.

**Table 2:** Circumstances in which OIB occurred and their Concurrent Behaviors according to the Caregiver Evaluation in Individuals with Autism (N = 74).

Circumstance(s) of occurrence	Concurrent behavior(s) occurring just before the OIB
Frustration40 (54%)	Anxiety-related behavior11 (14.9%)
Opposition28 (37.8%)	Excitation11 (14.9%)
Anger26 (35.1%)	Anger3 (4%)
Mainly in the presence of people27 (36.5%)	Self injurious behavior5 (6.7%)
Conflict24 (32.4%)	Tantrum5 (6.7%)
Mainly when the subject is alone10 (13.5%)	Verbal and non verbal language6 (8.1%)
Separation8 (10.8%)	Stereotypies3 (4%)
Isolation7 (9.4%)	Withdrawal0 (0%)

*Note:* Data are frequency of patients (% of group) for each circumstance of occurrence and concurrent behavior.

**Table 3:** Circumstances in which OIB Occurred and their Concurrent Behaviors according to the Parental Evaluation in Individuals with Autism (N = 74).

Circumstance(s) of occurrence	Concurrent behavior(s) occurring before the OIB
Frustration23 (31.1%)	Anxiety-related behavior18 (24.3%)
Opposition19 (25.7%)	Excitation9 (12.2%)
Anger20 (27%)	Anger4 (5.4%)
Conflict8 (10.8%)	Self injurious behavior2 (2.7%)
Mainly in the presence of people7 (9.4%)	Tantrum2 (2.7%)
Mainly when the subject is alone2 (2.7%)	Verbal and non verbal language1 (1.3%)
Separation2 (2.7%)	Stereotypies0 (0%)
Isolation0 (0%)	Withdrawal0 (0%)

*Note:* Data are frequency of patients (% of group) for each circumstance of occurrence and concurrent behavior.

According to the parental and caregiver observations, the most frequent emotional circumstances in which OIB occurred were frustration, anger and opposition. In addition, the most concurrent behaviors occurring just before the appearance of OIB in individuals with autism, were anxiety-related behaviors (such as anxious agitation, paleness, scream, sweat or facial expression of fear) and excitation (including joyful state) according to the parental as well as caregiver observation (see **Tables 2** and **3**).

### Comparative Study between individuals with autism and typically developing control individuals for OIB in the blood drawing situation

During the blood drawing situation, 15/64 (23%) individuals with autism showed OIB (biting, slapping, pinching) occurring in the minutes following the venepuncture. No OIB was observed in typically developing control individuals during the blood drawing even if certain children expressed their fear through verbal language and nonverbal behaviors (such as facial expressions). The comparative study between individuals with autism and typically developing control individuals controls for OIB occurring in the blood drawing situation showed that there was a significant and substantial difference in the distribution of OIB frequencies (χ^2^ (df = 1) = 30.38, *P*<.01): the observed frequency of OIB in individuals with autism was significantly higher than the expected values; inversely, the observed frequency of OIB in typically developing control individuals was significantly lower than the chance-based expected values.

## Discussion

The first main result of this study was the significantly lower OIB frequency (for any types of OIB) observed in children and adolescents with autistic disorder by parents at home compared to caregivers at day -care center. In addition, an absence of significant correlation between the parental and caregiver evaluations was found for the quantitative scores (Spearman correlations) of most of the types of OIB (see **Table 1**). We are confident in the validity of this result given the good inter-rater reliability (between two independent caregivers) reported for all the types of OIB (inter-rater agreement of 90%) except for the OIB “inserting objects into others' face orifices” [32], [33]. This result underlines also the interest of distinguishing different types of OIB for the descriptive and statistical analyses. These findings show the key role of the environment and more precisely of the situation and the observers in evaluations of OIB. I The higher OIB frequency (for any types of OIB) in children and adolescents with autistic disorder reported by caregivers compared to parents suggests that patients displayed more OIB in the day care center setting than at home. Indeed, it might be difficult for children with autistic disorder displaying social withdrawal to cope with a day care center environment involving group activities. Furthermore, several studies suggest that problem behaviors in children with developmental disabilities are often related to task demands [9]. It is possible that more demands were placed on the children in the day care center than at home. However, alternatively, this result might be related to a lower threshold of tolerance to OIB in caregivers compared to parents. Thus, the different results between the parental and caregiver observational situations underline the relational aspect that seems to exist in the expression of OIB. This relational aspect manifests itself (a) in the patient who modulates his/her behaviors as a function of the environment, i.e. the people who are around and the situation, and (b) in the observer who can perceive, interpret, and thus score differently OIB. It appears important to understand OIB in autism situationally, in the context of the relational dynamics arising between an individual expressing him or herself through a particular behavior related to a situation and an environment that perceives this particular behavior and responds to it with different tolerance thresholds according to the observers.

The second main result was the significantly higher frequency of OIB observed in children and adolescents with autistic disorder compared to typically developing control individuals during the blood drawing situation. Individuals with autism may perceive the blood drawing situation as more stressful than typically developing control individuals. This hypothesis is supported by our previous results showing enhanced biological stress responses to venipuncture in children with autism [26], [35]. In addition, children and adolescents with autistic disorder may be less capable than typically developing control individuals to regulate their emotional response to the blood drawing situation by expressing their noxious and/or psychic stress through verbal/non-verbal communication and social interaction, especially given their history of severe impairment in these two domains, and by developing other coping strategies. This result suggests that in a stressful situation, individuals with autism release their stress through OIB, whereas typically developing control individuals can regulate and express their stress using social interaction, verbal and non-verbal communication skills, as well as other cognitive skills such as symbolization skills with representation and anticipation of the stressful situation. This hypothesis is strengthened by our present results showing that the most frequent emotional circumstances in which OIB occur in autism are frustration, anger and opposition, and that the more frequent concurrent behaviors occurring just before OIB are anxiety-related behaviors and excitation. In addition, this hypothesis is in line with Dominick [25] who did not find any significant differences in OIB frequency between children with autistic spectrum disorder and children with history of language impairment. It is noteworthy that the usual decrease of aggression with age in typically developing children which begins at the age of two years old is explained, according to several authors, by the appearance of language [12], [36], [37]. Thus, the absence of significant age effect on OIB observed in individuals with autism from our study might be related to their verbal language impairment. Furthermore, the substantial and significant difference between individuals with autism and typically developing control individuals for the occurrence of OIB in the blood drawing situation might not be specific to autism but more related to mental retardation, especially if we consider that our autistic group is severely mentally retarded. However, given the very narrow range of IQ scores in the individuals with autism (IQ from 40 to 58), the relationship between IQ and OIB could not be thoroughly tested. Further studies taking into account the level of intellectual functioning are required in order to clarify this issue.

Finally, our results underline the important role of the environment, especially of the relational context, in the expression of OIB in children and adolescent with autistic disorder. Taken together with previous studies, they suggest also that OIB in autistic disorder is related to reactive aggression (impulsive and defensive aggression) in response to threat or frustration, and is not displayed without an environmental stimulus (including an imaginary threat) provoking psychic and/or physical stress with an emotional overload. This emotional overload would be released in autism through behaviors such as OIB and would not be regulated through cognitive skills such as social communication, symbolization skills or mental coping strategies. These findings could stimulate important research to study the relationship between anxiety and OIB and open new therapeutic perspectives on aggression in order to adapt the environment, decrease anxiety and better regulate stress responses in autistic disorder.
