# Correction: Aggression in Low Functioning Children and Adolescents with Autistic Disorder

**DOI:** 10.1371/annotation/45b0fa0e-071e-409e-be56-1004fcea0278

**Published:** 2011-03-09

**Authors:** Guillaume Bronsard, Michel Botbol, Sylvie Tordjman

There were errors in the Author Contributions. The correct contributions are: Conceived and designed the study: ST. Performed the study and contributed reagents/materials/analysis tools: ST MB GB. Analyzed the data: ST. Wrote the paper: ST GB.

